# Mycotoxins Contaminant in Kelp: A Neglected Dietary Exposure Pathway

**DOI:** 10.3390/toxins10110481

**Published:** 2018-11-19

**Authors:** Yanshen Li, Mingxue Sun, Xin Mao, Yanli You, Yonglin Gao, Jianrong Yang, Yongning Wu

**Affiliations:** 1 Marine Product Quality and Safety Inspection Key Laboratory in Shandong Province, College of Life Science, Yantai University, Yantai 264005, China; Sunmingxuee@outlook.com (M.S.); maoxin103820@ytu.edu.cn (X.M.) Youyanli@ytu.edu.cn (Y.Y.); gaoyonglin@ytu.edu.cn (Y.G.); yangjianrong@ytu.edu.cn (J.Y.); 2 NHC Key Lab of Food Safety Risk Assessment, China National Center for Food Safety Risk Assessment, Beijing 100022, China; 3 College of Food Science and Engineering, Shandong Agricultural University, Tai’an 271018, China

**Keywords:** LC-MS/MS, mycotoxins, dietary exposure risk, kelp, Northern China

## Abstract

In order to investigated current occurrence of major mycotoxins in dietary kelp in Shandong Province in Northern China, a reliable, sensitive, and rapid liquid chromatography tandem mass spectrometry (LC-MS/MS) method was developed and validated for simultaneous determination of the 7 most frequent mycotoxins, including 3-acetoxy deoxynivalenol (3AcDON), 15-acetoxy deoxynivalenol (15AcDON), Deoxynivalenol (DON), Fusarenon-X (F-X), Nivalenol (NIV), T-2 toxin (T-2), and Zearalenone (ZEA). Based on optimized pretreatment and chromatographic and mass spectrometry conditions, these target analytes could be monitored with mean recoveries from 72.59~107.34%, with intra–day RSD < 9.21%, inter–day RSD < 9.09%, LOD < 5.55 μg kg^−1^, and LOQ < 18.5 μg kg^−1^. Approximately 43 kelp samples were detected, 3AcDON/15AcDON ranged from 15.3 to 162.5 μg kg^−1^ with positive rate of 86% in Shandong Province in Northern China. Considering there were no related investigations about mycotoxin contamination in kelp, the high contamination rate of 3AcDON/15AcDON in kelp showed a neglected mycotoxin exposure pathway, which might lead to high dietary exposure risk to consumers.

## 1. Introduction

Mycotoxins are mainly produced by filamentous fungi in a complex matrix [1,2]. Mycotoxins can contaminate different agricultural commodities and they are mainly detected in cereals, such as barley, wheat, maize, and even fruit and related products [3,4,5,6]. Considering the severe toxicity, the presence of mycotoxins in foods could induce a high potential risk to human health, such as endocrine disorders, immunosuppression, teratogenic, carcinogenic and mutagenic effects, and so on [7,8]. In recent decades, due to the high frequency of contamination and widespread occurrence, mycotoxins have increasingly attracted attention worldwide.

It is well known the kelps are major keystone species which remain deep rooted in the marine environment [9]. Also, there are ample minerals and nutrients in kelps, which make them highly bioactive for human beings. Kelps usually grow on the bottom of the sea. They contain fiber, protein, beta carotene, amino acids, enzymes and chlorophyll, leading to the high quality in foods. In addition, there are also phosphorus, iron, sodium, potassium, calcium, magnesium, and other minerals in kelp [10]. Considering the similar components as cereals with protein and polysaccharose, fungi may also grow in kelps during the storage stage. Therefore, mycotoxins might also occur in kelps and related food and feeds. To the best of our knowledge, there is scarcely any information regarding the presence of mycotoxins in kelps. Reports about the transformation and generation of mycotoxins in kelps have also not been presented. In order to control mycotoxins in foods and feeds, the first and most important step is to develop sensitive and reliable methods for mycotoxin monitoring.

In the last decades, there have been numerous studies on mycotoxin detection with different chromatographic equipment, such as High Performance Liquid Chromatography (HPLC) [11,12,13], Liquid Chromatography Tandem Mass Spectrometry (LC-MS/MS) [14,15,16]), Gas Chromatography (GC) [17,18], Gas Chromatography Tandem Mass Spectrometry (GC-MS) [19,20,21,22], and so on. Antibody-based immunoassays were also applied for mycotoxin detection with advantages of simplicity, low–cost and high throughput. These immunoassays mainly include enzyme linked immunosorbent assay (ELISA) [23,24,25], fluorescence polarization immunoassay (FPIA) [26,27,28], surface plasmon resonance (SPR) [29,30,31,32], flow cytometric microsphere immunoassay [33,34,35], and rapid strip tests [36,37]. However, these methods mainly focused on cereal matrix and related products. As far as we know, there are very few reports on the detection of these targets in marine-derived products, especially kelps. Considering the high frequency of contamination of mycotoxins in cereals, exposure to kelps with similar components as cereals should be taken seriously. For the exposure investigation, the first and most important step is to develop a reliable detection method for mycotoxins in kelps.

In this work, a rapid, reliable, and sensitive LC-MS/MS method was developed for mycotoxin exposure detection in kelp. In order to obtain a satisfactory recovery for each analyte, sonication and an acidulated extraction pretreatment were investigated and optimized in this work. In order to minimize the matrix effect, each sample was further purified by a PLEXA cartridge. Based on this method, in 43 of 50 kelp samples in Shandong Province, 3AcDON/15AcDON was detected with a positive rate of 86%. In China, Shandong Province is one of the major kelp production and consumption areas, and the contamination of mycotoxins will lead to high dietary exposure risk to human beings.

## 2. Results and Discussion

### 2.1. LC-MS/MS Analysis

**Figure 1:**
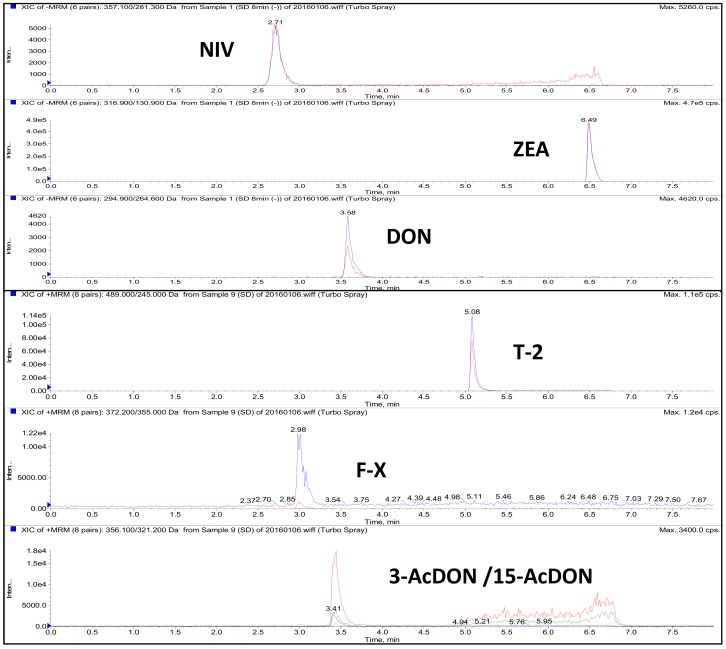
MRM chromatograms of 7 mycotoxins in kelp samples fortified at 50 μg kg^−1^.

The LC isocratic elution program for all 7 compounds could be finished within 7.5 min and resulted in satisfactory sensitivity and peak shape (Figure 1). From the figure, it is difficult to distinguish 3AcDON and 15AcDON from both the chromatogram and spectrum due to the similar structures and same precursor and production ions. Therefore, the two mycotoxins were monitored and calculated as a whole. The optimized MRM parameters are shown in Table 1. The result showed that DON, NIV, and ZEA exhibited higher response in ESI negative mode while 3AcDON/15AcDON, F-X, and T-2 toxin exhibited higher response in ESI positive mode, consistent with previous literature [38,39,40].

### 2.2. Optimization of Sample Preparation

#### 2.2.1. Optimization of Extraction Procedure

**Figure 2:**
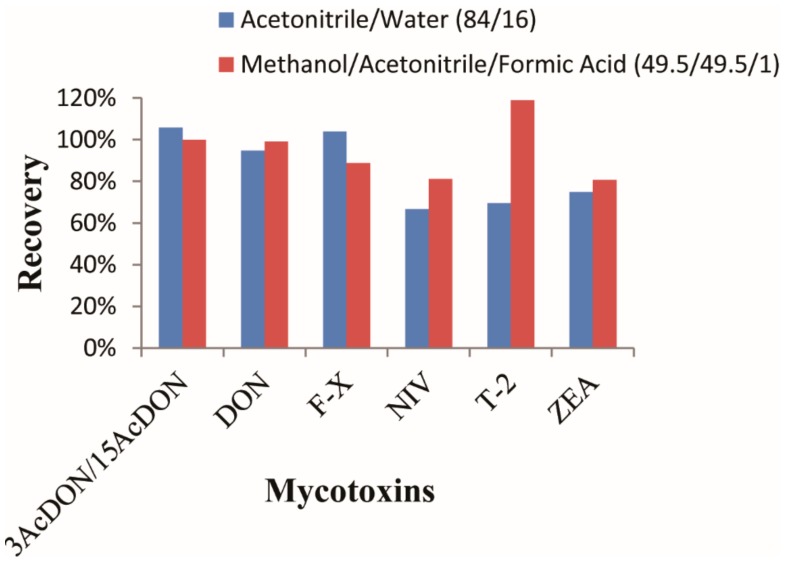
Optimization of extraction efficiency with two different extraction solvents.

Extraction is the first step in the sample preparation process for a satisfactory result. Methanol [41], ethyl acetate [42], acetonitrile and water [43] were adopted as the extraction solvents. For extraction of multiple mycotoxins, the most commonly used is Acetonitrile/water (84/16, *v*/*v*) [44,45]. According to the previous literature and the structures of these targets, different percentages of solvent were tested for extraction. The results showed that a single solvent (acetonitrile, ethyl acetate, methanol, and water) led to poor recovery for most target compounds. In this research, two combinations of acetonitrile/water (84/16, *v*/*v*) and methanol/acetonitrile/formic acid (49.5/49.5/1, *v*/*v*/*v*) were also investigated for extraction evaluation. Recoveries of target compounds applying the two extract solvents are shown in Figure 2.

From the figure, it could be concluded that recovery with acetonitrile/water (84/16, *v*/*v*) was better than that with methanol/acetonitrile/formic acid (49.5/49.5/1, *v*/*v*/*v*) for 3AcDON/15AcDON and F-X with recoveries all above 100%. This might be due to the high polarity of the three compounds. For the other mycotoxins, it was observed that methanol/acetonitrile/formic acid led to higher recoveries than acetonitrile/water. Especially for the T-2 toxin, obvious differences in recoveries were obtained between the two extract solvents, with methanol/acetonitrile/formic acid leading to a satisfactory result, while acetonitrile/water leading to a lower recovery. For DON, NIV, and ZEA, methanol/acetonitrile/formic acid exhibited higher recovery than the other solvents. Therefore, methanol/acetonitrile/formic acid (49.5/49.5/1, *v*/*v*/*v*) was adopted as the extraction solvent for all the seven mycotoxins.

For higher recovery, the extraction conditions were further optimized by sonication extraction time (1, 2, 3, and 4 min), formic acid concentration (0.5, 1, 1.5, and 2%), and volume of extract solvent (10, 15, 20, and 25 mL) for the extraction of 2 g of samples. The results are shown in Table 2.

From the table, it could be concluded that the most suitable concentration of formic acid for the extraction of all 7 mycotoxins was 1%. It was observed that 2% formic acid concentration led to a low concentration of DON, NIV, and ZEA, while 1.5% led to a low concentration of the T-2 toxin and DON. As for the concentration of formic acid at 0.5% and 1%, 1% concentration of formic acid led to a slightly better result. Therefore, 1% formic acid was adopted in this research. For the optimization of sonication, it was observed that 2 min and 4 min of sonication led to satisfactory recoveries of all the mycotoxins, while 1 min led to low recoveries of T-2 toxin, F-X, NIV, DON, and ZEA, and 3 min led to low recoveries of T-2 toxin and NIV. In order to simplify the extraction procedure, 2 min sonication time was adopted in this work. As for the volume of extract solvent for the extraction of 2-g samples, it was observed that 20 mL of extract could lead to satisfactory recoveries for all 7 compounds, while 10 and 15 mL of extraction solution was not sufficient to extract all the compounds. As for 25 mL of extract, low recoveries were obtained due to rapid loss of targets in the pretreatment process after excessive dilution.

#### 2.2.2. Optimization of Purification Procedure

**Figure 3:**
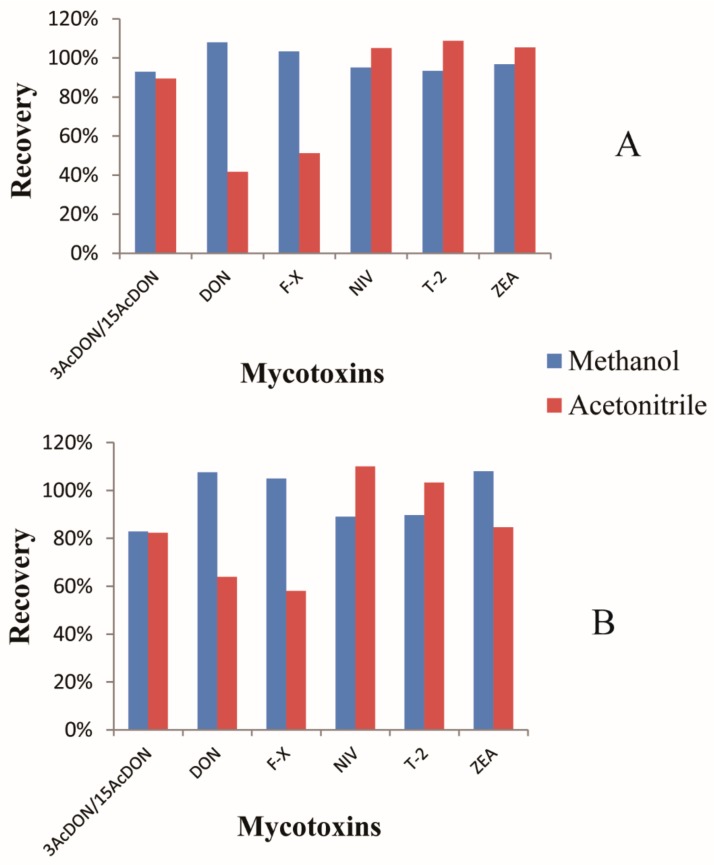
Optimization of the C18 cartridge with Methanol and Acetonitrile as the eluents (**A**). Optimization of the PLEXA cartridge with Methanol and Acetonitrile as the eluents (**B**).

In the previous, MSPD (matrix solid phase dispersion) [46,47], SPE (Solid Phase Extraction) [48], IAC (Immunoaffinity Colum) [6,11,43], and MycoSep227 [49] were applied for mycotoxin purification. However, MSPD requires a complicated treatment procedure and it is not suitable for high–throughput analysis. IAC requires specific antibodies for the combination of target analytes. Moreover, IAC is usually applied for high–specific and class–specific analysis of targets. It is not suitable for the detection of multiple mycotoxins. Therefore, in this research, SPE cartridges (C18 and PLEXA) were investigated and optimized with different elutions (methanol and acetonitrile) for the purification of the 7 target mycotoxins (Figure 3). From the figure, when acetonitrile was used as the elute solvent, recoveries of DON and F-X were very low. Recoveries of all the targets were satisfactory in both the PLEXA and C18 columns with methanol as the elute solvent. Furthermore, the recoveries of target analytes in the PLEXA column were higher than in the C18 column with methanol as the elute solvent. Therefore, the PLEXA column was adopted for mycotoxins purification with methanol as the elute solvent.

### 2.3. Method Validation

#### 2.3.1. Linearity 

In order to evaluate the linearity of this procedure, matrix-matched regression calibration curves were investigated at 7 spiked levels from 1 to 1000 μg kg^−1^. The equations for each analyte are shown in Table 3 with correlation coefficients (*r*) over 0.99. The wide range can cover the entire target analyte concentrations determined in clinical samples. Samples with high contaminated levels over liner range could be diluted before LC-MS/MS detection

#### 2.3.2. LOD (Limit of Detection)

LOD was determined on the basis of the S/N ratio higher than 3 in fortified samples, while LOQ (Limit of Quantification) was determined on the basis of the S/N ratio higher than 10. LOD and LOQ achieved in our work were sensitive. LOD was lower than 5.55 μg kg^−1^, while LOQ was lower than 18.5 μg kg^−1^ for all the analytes (Table 3).

#### 2.3.3. Accuracy and Precision 

Accuracy and precision of this method are shown in Table 4. These values were evaluated from recoveries of each analyte in fortified samples at two different spiked concentrations. Recoveries were determined by the calculated concentrations divided by the spiked levels. The intra–day and inter–day RSDs (Relative Standard Deviations) of each analyte were determined from process fortified samples with each concentration of 5 replicates on three separate days. From the table, mean recoveries were 72.59~107.34% for all the analytes with intra–day RSD less than 9.21% and inter–day RSD less than 9.09%, respectively. From the results, it can be concluded that the developed method could be applied to monitor these mycotoxins in kelps.

### 2.4. Dietary Exposure of Mycotoxins in Kelp

In order to investigate the dietary exposure of mycotoxins in kelp, 50 kelp samples were obtained from a local supermarket in Shandong Province, China. Each sample was processed according to this LC-MS/MS protocol. In total, 43 kelp samples were detected with 3AcDON/15AcDON, with a positive rate of 86%, while T-2, F-X, DON, ZEA and NIV were negative in all tested samples in Shandong Province in Northern China (Table 5). Considering that the major difference between kelps and cereals is salinity, it seems that the Foodborne fungi can mainly produce acetylated metabolites of DON. As for T-2 toxin, F-X, ZEA, and NIV, they might not be produced in marine food with a high percentage of salinity. It is reported that the kelp production in China accounts for almost 80% of the whole world. In China, Shandong Province, Fujian Province, and Liaoning Province are the main kelp production regions, which produce about 99% of the total kelp output, which is over two million tons (Analysis and prospect of kelp industry development in China, 2016–2020). The kelp production in Shandong Province is over 800 thousand tons. The consumption patterns mainly include direct consumption and health product production. However, investigations about mycotoxins in kelp are limited until now. The exposure risk of mycotoxins in kelp dietary is neglected. From the commercial samples tested in this work, it can be concluded that dietary kelp might be a potential exposure pathway of 3AcDON/15AcDON.

## 3. Conclusions

In conclusion, a sonication based quantitative and confirmatory LC-MS/MS procedure was developed for the determination of 7 major mycotoxins (3AcDON, 15AcDON, DON, F-X, NIV, T-2, and ZEA). Specifically, target analytes were extracted with acidulated methanol/acetonitrile/formic acid (49.5/49.5/1, *v*/*v*/*v*). After the extraction, each sample was further purified by a PLEXA cartridge to minimize the matrix effect. The validation of this developed procedure proved the suitability of the method for the confirmatory analysis of mycotoxins with mean recoveries from 72.59~107.34%, intra-day RSD < 9.21%, inter-day RSD < 9.09%, LOD < 5.55 μg kg^−1^, and LOQ < 18.5 μg kg^−1^. With respect to real samples, T-2, F-X, DON, ZEA and NIV were not detected in any sample, while all samples had 3AcDON/15AcDON that ranged from 57.5 to 162.5 μg kg^−1^ with a positive rate of 86%. Considering that Shandong Province is one of the major kelp production and consumption areas in China (over 40%), the contamination of mycotoxins will lead to high dietary exposure risk to human beings.

## 4. Materials and Methods

### 4.1. Chemicals and Reagents

**Figure 4:**
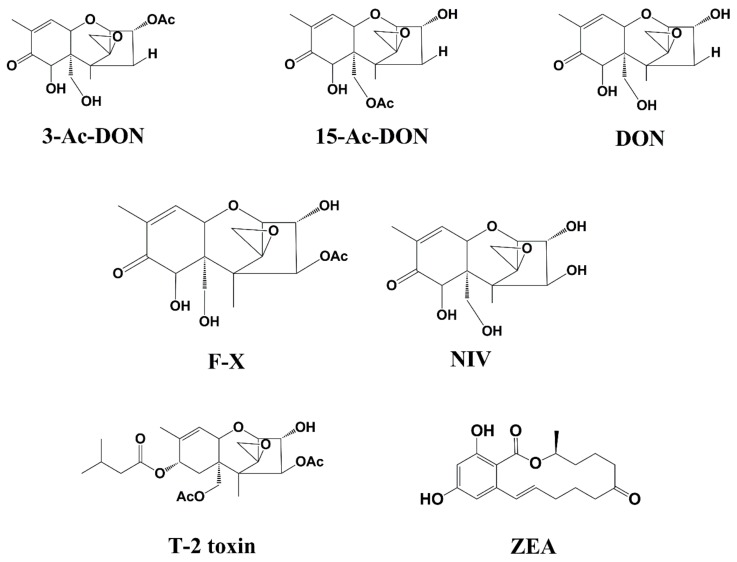
Chemical structure of 3-AcDON, 15-AcDON, DON, F-X, NIV, T-2 toxin, and ZEA.

3-Acetyldeoxynivalenol (3AcDON), 15-Acetyldeoxynivalenol (15AcDON), Deoxynivalenol (DON), Nivalenol (NIV), T-2 toxin (T-2), and Zearalenone (ZEA) (Figure 4) were obtained from Fermentek Biotechnology (Jerusalem, Israel).

Acetonitrile and methanol (HPLC) were adopted in this work (Dima Technology Inc.) (Muskegon, MI, USA). Formic acid (HPLC) was purchased from Fisher Scientific Inc. (Pittsburgh, PA, USA). Milli–Q Synthesis system (Millipore, Bedford, MA, USA) was used for water purification. Bond Elut PLEXA cartridges (500 mg, 6 cc) (Agilent Technologies, CA, USA) were used in this work. Other reagents were obtained from Sinopharm Chemical Reagent Beijing Co., Ltd. (Beijing, China).

### 4.2. Apparatus

The LC system adopted in this research was obtained from AB SCIEX (Redwood City, CA, USA) with a Venusil ASB C18 column (100 mm × 2.1 mm i.d., 3 μm particle size). The quadrupole mass spectrometer used in this work was a AB4000 triple from AB SCIEX (Redwood, CA, USA). The vortex mixer was from North TZ–Biotech Develop Co., Ltd. (Beijing, China). The N-EVAP 112 nitrogen evaporator was from Organomation Associates (Berlin, MA, USA).

### 4.3. Sample Preparation

Two grams of dried kelp sample was weighed into a 50-mL polypropylene centrifuge tube. Two experiment groups were fortified with 50 and 100 µg kg^−1^ of each analyte. One unfortified group was set as the negative control. Twenty milliliters of methanol/ethyl acetate/formic acid (49.5/49.5/1, *v*/*v*/*v*) was added and ultrasound was performed for 2 min followed by vortexing for 3 min for extraction. Each sample was centrifuged at 9000 rpm for 10 min at 4 °C. The supernatant was transferred and dried using a nitrogen evaporator at 60 °C. The residues were re-dissolved with 10 mL of water by vortexing for 3 min. Each sample was loaded onto a PLEXA cartridge with 5 mL of methanol and 5 mL of water in turn. After rinsing with 5 mL of water, analytes were eluted with 5 mL of methanol. After drying using a nitrogen evaporator at 60 °C, target analytes were re-dissolved with 1 mL of water/acetonitrile(9/1, *v*/*v*). Samples were filtered through a 0.22-μm filter and 10 μL was injected for LC-MS/MS analysis.

### 4.4. Instrumental Conditions

Target analytes were separated via LC system with Venusil ASB C18 column. The mobile phase was as follows: solvent A (water containing 50 μM of ammonium acetate) and solvent B (acetonitrile). The column temperature was set to 25 °C, and the flow rate was 0.5 mL min^−1^ with injection volume of 10 μL. The gradient elution program was performed for chromatography separation as follows: 0–1 min, 98% A; 1 to 3 min, 98–60% A; 3.0–4.0 min, 60% A; 4.0–5.0 min, 60–10% A; 5.0–6.1 min 10% A; 6.1–6.2 min 10–98% A; 6.2–8.0 min 98% A.

For detection, the LC system was coupled to an AB4000 triple quadrupole mass spectrometer (Redwood, CA, USA) with an electrospray ionization source (ESI). For maximum intensity detection, the mass conditions were optimized as follows: Capillary voltage at 5.0 kV; Source temperature at 550 °C, IonSpray voltage at 5500 V. Ion Source Gas 1 was 55 Psi, and Ion Source Gas 2 was 55 Psi. The MS instrument was operated in integrate ESI positive (ESI+) and negative (ESI−) multiple reaction monitoring (MRM) mode (Table 1).

## Figures and Tables

**Table 1:** MRM parameters of 7 mycotoxins.

Analyte	Scan Mode	Precursor Ion (*m*/*z*)	Product Ion (*m*/*z*)	Cone Voltage (V)	Collision Energy (eV)
3AcDON/15AcDON	ESI+	356.2	339.1	55.99	20.00
			321.2 *	45.00	18.73
			230.9	55.99	23.80
			145.3	45.00	25.92
DON	ESI−	294.9	264.6 *	−70.89	−15.12
			137.9	−69.02	−24.18
F-X	ESI+	372.2	355.0 *	4.87	10.78
			247.3	7.90	19.02
NIV	ESI−	357.1	311.3	−54.95	−16.25
			281.3 *	−54.95	−12.90
T-2	ESI+	489.0	327.0	20.00	245.00
			245.0 *	20.00	327.00
ZEA	ESI−	316.9	175.0	−80.00	−39.82
			130.9 *	−80.00	−34.62

* (ion for quantification).

**Table 2:** Optimization of different extraction conditions.

Condition	Parameter	Recovery					
		3AcDON/15AcDON	T-2	F-X	NIV	DON	ZEA
Formic Acid (%)	0.5	82%	95%	78%	79%	9%	91%
	1	91%	90%	91%	77%	94%	96%
	1.5	90%	72%	80%	79%	90%	95%
	2	91%	99%	86%	72%	74%	77%
Sonication (min)	1	89%	75%	80%	82%	63%	74%
	2	95%	91%	90%	94%	101%	81%
	3	95%	75%	92%	82%	101%	86%
	4	100%	90%	91%	81%	99%	88%
Extract Volume (mL)	10	91%	80%	90%	68%	80%	93%
	15	98%	102%	80%	70%	84%	77%
	20	102%	99%	94%	84%	90%	91%
	25	95%	101%	96%	69%	95%	90%

**Table 3:** Parameters (including the standard curve, LOD, and LOQ) of 7 mycotoxins in kelps.

Analyte	Liner Range (μg kg^−^^1^)	Regression Equitation	*r*	LOD (μg kg^−^^1^)	LOQ (μg kg^−^^1^)
3AcDON/15AcDON	1.0–1000	*y* = 17.55*x* + 5730.2	0.9981	3.02	10.06
DON	1.0–1000	*y* = 24.62*x* + 217.57	0.9994	2.6	8.68
F-X	1.0–1000	*y* = 19.84*x* + 3384.8	0.9902	5.55	18.5
NIV	1.0–1000	*y* = 2.016*x* + 439.40	0.9931	1.14	3.81
T-2	1.0–1000	*y* = 46.75*x* + 76.992	0.9921	0.16	0.53
ZEA	1.0–1000	*y* = 399.4*x* + 178.21	0.9949	0.22	0.73

**Table 4:** Accuracy and precision of mycotoxins in kelps.

Analyte	Spiked Level (μg kg^−^^1^)	Day 1		Day 2		Day 3		Inter-Day RSD % (*n* = 15)
		Mean Recovery (%)	Intra-Day RSD % (*n* = 5)	Mean Recovery (%)	Intra-Day RSD % (*n* = 5)	Mean Recovery (%)	Intra-Day RSD % (*n* = 5)	
3AcDON/15AcDON	50	90.21 ± 2.71	2.81	91.46 ± 1.04	1.04	93.13 ± 6.25	5.97	3.65
	100	92.59 ± 9.26	9.17	101.5 ± 8.52	7.77	91.11 ± 8.89	9.21	9.09
DON	50	75.61 ± 2.05	2.41	77.08 ± 0.58	0.71	79.83 ± 3.26	4.07	3.41
	100	101.1 ± 4.01	3.76	101.2 ± 7.33	7.08	104.9 ± 3.33	3.07	4.63
F-X	50	97.50 ± 0.83	0.85	98.61 ± 3.06	2.72	101.1 ± 1.94	1.71	2.32
	100	98.50 ± 5.51	5.01	94.33 ± 5.67	5.44	93.80 ± 8.7	8.06	5.91
NIV	50	104.0 ± 4.63	3.86	103.0 ± 1.67	1.48	107.3 ± 5.65	4.82	3.71
	100	93.97 ± 6.83	6.31	93.98 ± 7.22	6.68	99.88 ± 9.69	9.12	7.23
T-2	50	91.23 ± 6.23	6.45	90.00 ± 5.01	4.84	94.37 ± 2.73	2.72	4.73
	100	97.38 ± 9.76	9.05	89.52 ± 8.09	8.79	90.83 ± 9.4	8.98	8.71
ZEA	50	72.59 ± 0.74	0.88	74.44 ± 5.22	3.95	80.00 ± 4.44	4.81	5.47
	100	86.24 ± 7.09	7.46	77.65 ± 5.68	6.34	77.51 ± 3.26	3.82	7.59

**Table 5:** Mycotoxin contamination exposure in commercial kelp samples in Shandong Province in Northern China.

No.	3AcDON/15AcDON (μg kg^−^^1^)	Other Mycotoxins (μg kg^−^^1^)	No.	3AcDON/15AcDON (μg kg^−^^1^)	Other Mycotoxins (μg kg^−^^1^)	No.	3AcDON/15AcDON (μg kg^−^^1^)	Other Mycotoxins (μg kg^−^^1^)	No.	3AcDON/15AcDON (μg kg^−^^1^)	Other Mycotoxins (μg kg^−^^1^)
1	100	ND	14	25.6	ND	27	15.3	ND	40	54.8	ND
2	87.5	ND	15	ND	ND	28	19	ND	41	36.1	ND
3	57.5	ND	16	ND	ND	29	ND	ND	42	ND	ND
4	75	ND	17	35.9	ND	30	39.3	ND	43	27.4	ND
5	137.5	ND	18	41.2	ND	31	42.1	ND	44	58	ND
6	78.75	ND	19	31.9	ND	32	ND	ND	45	37.5	ND
7	106.25	ND	20	28.5	ND	33	21.6	ND	46	55.3	ND
8	77.5	ND	21	22.5	ND	34	33.9	ND	47	46.7	ND
9	162.5	ND	22	ND	ND	35	58.9	ND	48	43.1	ND
10	87.5	ND	23	43.8	ND	36	68.3	ND	49	28.9	ND
11	118.75	ND	24	56.7	ND	37	17.3	ND	50	36.2	ND
12	96.3	ND	25	55.2	ND	38	22.8	ND			
13	33.2	ND	26	21.6	ND	39	ND	ND			

Other mycotoxins include DON, F-X, NIV, T-2, and ZEA.
